# Understanding specific gender dynamics in the cowpea value chain for key traits to inform cowpea breeding programs in Malawi, Mozambique and Tanzania

**DOI:** 10.3389/fsoc.2024.1254292

**Published:** 2024-02-15

**Authors:** Michael M. Chipeta, Jessica Kampanje-Phiri, Dumisani Moyo, Henriques Colial, Mussa Tamba, Divage Belarmino, Joseph Hella, Esnart Yohane, Naomi Mvula, John Kafwambira

**Affiliations:** ^1^Lilongwe University of Agriculture and Natural Resources, Lilongwe, Malawi; ^2^Development with Data Science (DDSc), Lilongwe, Malawi; ^3^Instituto De Investigação Agrária De Moçambique, Nampula, Mozambique; ^4^Tanzania Agriculture Research Institute, Illonga, Tanzania; ^5^Sokoine University of Agriculture, Morogoro, Tanzania; ^6^Department of Agricultural Research Services, Chitedze Research Station, Lilongwe, Malawi

**Keywords:** cowpea value chain, gender sensitive cowpea breeding, priority traits, product profiles, trait preferences

## Abstract

**Introduction:**

Cowpea is an important food and nutrition security crop in Malawi, Mozambique and Tanzania and it is mainly produced by women farmers mainly on a subsistence scale. The majority of these farmers use local varieties despite the availability of improved varieties in the region. Low acceptability and adoption of improved varieties have also hampered cowpea breeding efforts. The low adoption, especially among women farmers, has been attributed to the failure by breeding programs to involve farmers in the process of designing and developing improved varieties with a view to meeting their priorities and preferences. Despite women constituting the majority of cowpea farmers in these countries, no comprehensive gender analysis on cowpea value chain had been instituted to understand the traits that are gender and youth responsive and how to incorporate them in the product profiling so that the developed varieties benefit men, women and youth. The main objective of the gender study was, therefore, to identify preferred traits by different gender groups within the whole cowpea value chain to inform cowpea breeding programs in the three countries.

**Methods:**

The study employed quantitative and qualitative methods to elucidate preferences, including value chain mapping, a quantitative survey of farmers, focus group discussions and key informant interviews targeting farmers/consumers, traders, policymakers and processors.

**Results and discussion:**

Results showed that the top-ranking traits in order of importance across the countries and gender were; (1) high grain yield, (2) good grain taste, (3) early maturity time, (4) large grain size, (5) good leaf taste and (6) short cooking time. It was further noted that different gender groups preferred almost similar traits though minor variations were noted in terms of prioritization of these traits. These results have had two major influences on our cowpea breeding program: firstly, the breeding program changed the way it prioritizes traits to include ones that reflect the needs of men, women and the youth in the cowpea value chain. Secondly, our breeding objectives are closely aligned to gender differences in the target population of farmers and other users, by incorporating key priority traits that address the needs of both men and women, including the youth. That is to say, product targets and specific product profiles are more gender sensitive. Since the breeding work is ongoing, the expectation is that the development of improved varieties resulting from this gender sensitive process will translate into higher adoption levels of these varieties (compared with previous releases), which might have ripple effects on food, nutrition and income security in the region.

## 1 Introduction

Cowpea (*Vigna unguiculata* (L.) Wallp.) is a leguminous crop that is grown in most parts of the world but it is mainly produced and consumed in sub-Saharan Africa (Boukar et al., 2019). Most of the production in sub-Saharan Africa is by smallholder farmers, especially women in marginal conditions, often as an intercrop with maize, sorghum, or millet (Ehlers and Hall, 1997). Cowpea is one of the primary sources of inexpensive, high-quality protein and micronutrients (Fe, Ca, Zn), and thus, it can positively impact the health of men, women and children (Nielsen et al., 1997; Thomson, 2008; Nelson et al., 2008; Carsky et al., 2010; Boukar et al., 2011, 2016; Muñoz-Amatriaín et al., 2017). Cowpea is important to the nutrition and income of smallholder farmers, especially women and youth in Malawi, Mozambique and Tanzania, and it also contributes to the sustainability of the cropping system through fixation of atmospheric nitrogen and prevention of soil erosion.

Malawi, Mozambique and Tanzania national breeding programs in collaboration with other partners, had in the past released improved cowpea varieties to address food and nutrition security. However, the majority of the farmers still grow unimproved varieties/local cowpea −90% in Malawi, 89.1% in Mozambique and 68.7% in Tanzania (IFPRI, 2020). Like in many parts of Sub-Saharan Africa (Murdock et al., 2013), cowpea in Malawi, Mozambique and Tanzania is primarily produced by women as well as the youth as a source of food and income. It was unclear whether the reported low adoption rates reflected the fact that the varieties released in these countries do not meet women and youth preferences. Earlier studies (Chiulele et al., 2011; Hella et al., 2013) in Malawi, Mozambique and Tanzania showed that farmers preferred cowpea with large grain size, brown colored grains, early maturity and high grain yield among others. These studies, however, did not collect gender disaggregated data on trait preferences. However, men and women may have different reasons for adopting a new crop variety as also echoed by Polar et al. (2017) that agricultural technologies are not gender neutral. What was very clear, however, at the onset was that the national breeding programs in these countries, in the past, failed to involve women and youth farmers in the process of designing and developing improved varieties with a view of meeting their priorities and preferences. Plausibly this led to low adoption of improved varieties. Therefore, it was imperative to conduct a comprehensive gender analysis on the cowpea value chain to understand the traits that are gender and youth responsive and how to incorporate them in product profiling so that varieties developed and released benefit men, women and youth. To address this gap, Lilongwe University of Agriculture and Natural Resources (LUANAR), Instituto de Investigação Agrária de Moçambique (IIAM) and Tanzania Agriculture Research Institute (TARI-ILONGA) joined to form the Center of Innovation for Crop Improvement for East and Southern Africa (CICI-ESA). The center also works together with the Department of Agriculture and Research Services in Malawi and Sokoine University of Agriculture in Tanzania. The CICI-ESA breeding program prioritizes the inclusion of male, female and young farmers in Malawi, Mozambique and Tanzania in the process of designing and developing more productive and nutritious cowpea varieties. As such, the team conducted a comprehensive gender-sensitive cowpea value chain analysis in the three countries to understand specific gender and youth dynamics in the cowpea value chain. Specifically the study was conducted to: (i) understand the role of men, women, and youth within the whole Cowpea value chain, (ii) identify preferred traits by different gender groups within the whole cowpea value chain to inform cowpea breeding programs, and (iii) map out cowpea markets to identify core processes of the value chain, the enabling environment, value chain actors, service providers, market information, financial services, and transport services that would inform other cowpea marketing opportunities for both small scale and large scale farmers.

## 2 Materials and methods

### 2.1 Study sites/locations

**Figure 1:**
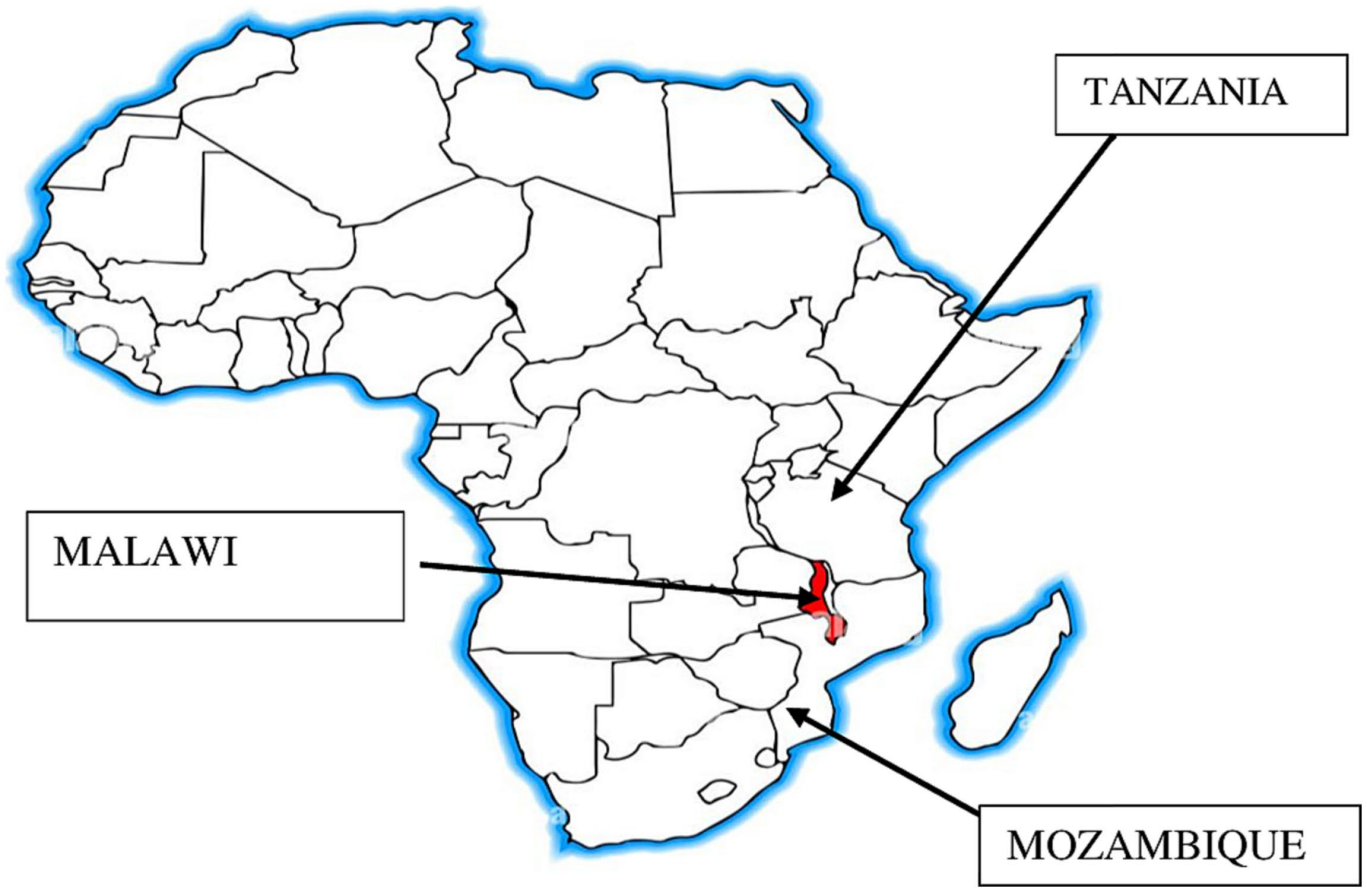
Map showing study countries for the gender sensitive value chain analysis study.

This study was conducted in three countries namely, Malawi, Mozambique and Tanzania (Figure 1). Specifically, in Malawi, the study was implemented in six cowpea growing districts (two districts per region) namely, Chikwawa and Mulanje, (southern); Dedza and Salima (central); Karonga and Mzimba (northern). Similarly, six cowpea growing districts were sampled as study sites across three provinces of Mozambique, namely, Rapale, Mogovolas, and Meconta (Nampula); Ancuabe and Chuire (Cabo Delgado); Alto Molocue (Zambezia). Finally, in Tanzania, six districts were also sampled across three regions namely, Kongwa and Bahi (Dodoma); Kilosa and Mvomero (Morogoro); Iringa Rural and Kilolo (Iringa). The selected districts within each country were selected for the study because they are the major cowpea producing areas in each region/province of each country.

### 2.2 Team composition of the gender study

A multi-disciplinary team from the three countries championed the gender analysis study, each with specific roles. The research team was led by breeders who wanted to understand the sociocultural contexts of running a breeding program that integrates specific gender dynamics and whether these dynamics can influence the prioritization of traits, formulation of breeding objectives and defining product profiles. Breeders played a key role in the gender study through identification of study areas where cowpea is mainly grown, defining and explaining the different cowpea traits as well as leading in product profile development. Social scientists (gender experts, social economists, nutritionists, and sociologists), were at the core of operationalizing a cowpea-gendered study under two themes; ‘priority setting and crosscutting issues’. Social economists led the design of the quantitative methodologies, which included trait preference surveys (i.e., sampling design including sample size, sampling framework and survey tools). Gender experts led the design and analysis of the qualitative methodologies (value chain mapping, focus group discussions and key informant interview guides).

### 2.3 Trait preference survey/gender sensitive value chain analysis

The study followed a gender-sensitive value chain mapping approach which is a process that includes both quantitative and qualitative data collection. The quantitative-engendered mapping exercise helps researchers to determine labor allocation, returns, and ownership along the chain, amongst other parameters, while the qualitative mapping exercise complements the quantitative data by collecting data on the factors that shape particular outcomes for men and women along the chain (USAID, 2009).

### 2.4 Quantitative survey design and sampling procedure

A multi-stage sampling technique was used to select a sample of farm households as described below randomly:

**Stage 1: identification/selection of survey sites**–the districts (6 districts within the 3 regions/provinces of each country-total of 18 districts) were selected by critically assessing the Agricultural Production Estimates (APES) data, and those districts where cowpea is mostly grown were purposively selected. However, the selection of the focal sites within each of the selected districts was done in liaison with Agricultural Officers as well as specific consideration context with regards to land inheritance systems such as patrilineal and matrilineal predominance.

**Stage 2: random selection of sample villages clusters**. This was done from a listing of villages within the selected survey sites, using a simple random sampling technique.

**Stage 3: random selection of sample households using lists of farm households involved in the cowpea value chain.** The entry point to each study site within each district was the Agricultural Officers who have the knowledge and lists of farmers involved in cowpea value chains. These lists summarized by the village were used to select farm households randomly.

**Sample sizes;** actual sample size for farm households was determined scientifically, using Cochran’s (1963) formula for determining a representative sample for a large population:


$$n=\frac{Z^{2}pq}{e^{2}}$$

where n is the sample size, Z is the chosen Z-score corresponding to the desired confidence level (i.e., 95%), e is the desired level of precision (i.e., 5%), and p is the estimated proportion of an attribute that is present in the population (50% is adopted to obtain maximum sample size), and q is 1-p. This yielded sample size of 385. Taking into consideration for no-responses, the final sample size was 396 per country (66 per district). However, for Malawi and Mozambique the sampled farmers were more than estimated and in Tanzania it was less than estimated (Table 1).

**Table 1:** Total number of farmers sampled for gender analysis study.

Malawi		Mozambique		Tanzania	
Region/District	Total sampled	Region/District	Total sampled	Region/District	Total sampled
*Northern region*		*Nampula province*		*Dodoma region*	
Karonga	91	Rapale	77	Kongwa	40
Mzimba	66	Mogovolas	72	Bahi	40
		Meconta	68		
*Central region*		*Cabo Delgado province*		*Iringa region*	
Salima	68	Ancuabe	81	Iringa Rural	40
Dedza	73	Chuire	71	Kilolo	40
*Southern region*		*Zambezia province*		*Morogoro region*	
Mulanje	75	Alto Molocue	74	Kilosa	40
Chikwawa	75			Mvomero	40
Total sampled	448		443		240

### 2.5 Qualitative survey design

In line with our gender-sensitive value chain analysis focus as outlined above, qualitative data collection was done at different levels targeting male and female cowpea farmers; lead farmers, community leaders, agro-dealers, extension workers, traders, policymakers, processors, amongst others. The data collection at these levels combined various methods, including, Value Chain Mapping, Key Informant Interviews (KIIs), Focus Group Discussions (FGDs), and Observations.

#### 2.5.1 Cowpea value chain mapping

Value chain mapping with stakeholders was meant to understand and identify main cowpea players and their roles, mapping various segments of the value chain, identifying main products and processes and mapping out preferred cowpea traits within the different nodes of the cowpea value chain. This activity helped the team have a bigger picture of who is involved at each node of the value chains and aided in refining the proposed research approach. This exercise also helped to contextualize and validate the literature review and streamline the research focus of gender assessment in general. The value chain mapping exercise was split into three activities, which were done sequentially as follows:

- **a.Participatory value chain mapping**

This was done together with selected gender group representatives involved in the cowpea value chain at a specific level (i.e., village, community, and district). Specifically, each value chain mapping group ideally was comprised of cowpea farmers/consumers (10 male; 10 female; 5 male youth; 5 female youth), 5 male/5 female (traders and agro-dealers representatives where present), agriculture extension workers and district agriculture officers where present. Some of the activities involved during this process included:

- Preparation of the value chain maps together with the actors present to identify relevant categories for the value chain actors.
- Maps laid out on flip charts by use of a checklist and used to map out actors, activities, flows/relationships, and contexts for cowpea within each context.
- The process of developing the map was iterative until there was consensus on the text and visual narratives about the value chain actors, how they are networked, and how the resources flow between them.

- **b.Understanding horizontal and vertical value chain integration**

Once actors and activities for each value chain were mapped, specific flows/relationships in the cowpea value chain were established. This process assisted in clarifying the linkages further by tracing in what ways the actors, goods/products, or activities are mainly flowing. Are they happening at the same level or at various levels? Thus, the overall aim of this step was to explain vertical/horizontal value chain integration, i.e., the actors (the people), the linkages (the relationships), and the flows (how goods and services move from one actor to another through linkages). This process also assisted the research team to clarify any existing gender differences in terms of actors and activities involved in each node of the cowpea value chain as well as coming up with crude percentage estimates of gendered actors involved at each identified node of the value chain.

- **c.Identifying gendered barriers and leverage points for change**

This last step aimed to ask participants to validate the map, make revisions if necessary, and use it as a boundary object for discussing gendered constraints and opportunities along the value chain. This step was critical as it gave both the research team and the stakeholders involved a snapshot of how a specific value chain operates and aided to either modifying study tools or contextualize some issues further as captured by the value chain map. Through this process, study tools were also further pilot tested in Mozambique before sharing them to the other CICI-ESA countries since the gender sensitive value chain studies were done consecutively within the different counties in question.

The value chain mapping processes were adapted from a *Developing gender-sensitive value chains – a guiding framework* (FAO, 2016) and a gender value chain toolkit by Laven and Verhart (2011).

#### 2.5.2 Focus group discussions

The study had standard focus group guiding figures that were used or modified based on the availability of the groups for each of our study sites as follows: 6 FGDs with farmers (3 adult male/3 adult females), 6 FGD with male and female youth farmers where available; 1 FGD with lead farmers per region/province (not disaggregated by gender, but based on availability), 1 FGD with community leaders per region/province (not disaggregated by gender, but based on availability). Interview guides with semi-structured questions were used to guide discussions and the gathering of appropriate data from these sources. Key focus issues during focus group discussions were: differences between men and women in terms of land ownership, who commonly makes the decision on cowpea production or which varieties to grow in such households and why? criteria that men and women apply when deciding to try or adopt new cowpea varieties, who does what during cowpea production, what cowpea traits/varieties were preferred by each gender group and why, etc.

#### 2.5.3 Key informant interviews and observations

Key Informant interviews with 10–15 agro-dealers, 6 extension workers and at least 3 policymakers were also conducted for all the three countries. Therefore, the research team facilitated participatory and consultative sessions, including individual interviews (IDIs), KIIs and focus group discussion sessions across the study sites.

### 2.6 Data collected in relation to gender

The gender analysis study focused on the following key issues:

Trait preference data on farmers and consumers were collected according to gender category classified as adult male, adult female, young male and young female. Here, farmers and consumers were asked to indicate the level of importance (on a scale of 4 to 1, denoting, not important, less important, important, very important, in that order) that they attach to a particular trait, the varieties they prefer and their attributes which determine their choice in terms of production or utilization.

Apart from farmers and consumers, we also engaged agro-dealers, traders, and processors. Agro-dealers were engaged to learn on the different inputs stocked related to cowpea production, such as the availability of improved seed, pesticides, agricultural implements and storage materials used in cowpea and whether their main customers are men or female. Traders were also a central focus of this study to understand the movement of cowpea within the value chain, the type of traders (male or female), the kind of cowpea varieties they prefer to buy and whether female and male consumers have different preferences to certain varieties or not. For processors, we focused on the types of varieties processors use and their attributes, their main suppliers, main products and by-products from cowpea processing and the target markets (whether local or export markets) with a gender lens. Throughout the qualitative data collection processes, the research team was also encouraged to record any observations related to the research issues in questions. Table displays an example of country summary for types of data collected based on the methodologies used under this study.

### 2.7 Data analysis

Data was analyzed separately for each country. Quantitative data was analyzed using SPSS (version 25.0) and STATA (version 17) to generate frequencies, descriptive statistics, cross-tabulations, and chi-square tests. For qualitative data, Kabeer’s Social Relations Framework approach was adopted to examine gender dynamics within domains like household, community, state, and markets. This Framework as a gender analytical tool allows an exploration of gender relations from problem conceptualization to analysis of data in different spheres of social enquiry. The framework also emphasizes the subjective meanings of empowerment and the pathways to it, and the need to understand the context-specific and culturally embedded nature of gender relations (Hillenbrand et al., 2014). In this study, this framework was used to frame our overall gender objectives and approach of the comprehensive gender analysis within the cowpea value chain. Thus, by looking at how different concepts such as agency, structure, and discourse influence or are related to specific gendered behaviors or practices. This framework allowed us to take a value chain approach to examine gender dynamics within different domains and spheres as specified above. As such, the social relations between different actors, such as men and women, boys and girls and how these relations shape their access to resources, opportunities, and decision-making powers, were some of the core analyses that took place in all our study sites guided by this framework.

Additionally, content analysis, thematic analysis, grounded theory, and ethnographic analysis were used to identify cowpea value chain actors, their roles, emerging themes and constraints/opportunities related to cowpea value chain and gender (see also, Vaismoradi et al., 2013). In this case, content analysis involved coding and categorizing our qualitative data from the FGDs and KIIs into themes, concepts and categories, based on predefined topics in our interview guide as well as from emergent themes. For example, a theme on ‘cowpea trait preference’ was further categorized by gender and comparable across gender groups based on both a predefined criteria and the emerging data that was showing differences in preferences between male, female and young cowpea farmers. Similarly, thematic analysis in this study also involved identifying, analyzing, and reporting the patterns or themes that emerge from our data, and relating these themes to our overall research questions. For example, common or recurring issues, such as male farmers’ preferences on cowpea traits that had potential market viability could be traced across districts within countries and across all our study sites. This analytical method therefore helped us to generalize patterns of behaviors within and across specific gender groups as shown in our results. Grounded theory being another analytical tool that we used allowed us to further analyze the common patterns into theoretical assumptions that allowed us to compare and contrast different behaviors of cowpea value chain actors along the value chain. For example, this iterative process allowed us to move across different types of data collected in our study (i.e., quantitative and qualitative) and make comparisons on emerging issues. This process also helped a lot in coming up with specific recommendation of trait preferences for specific gendered groups based on commonalities or differences identified through this iterative process. Finally, ethnographic analysis in this study was further used to understand themes or issues with reference to culture, norms, and practices of the specific gendered groups in our study. Thus, through participatory observation, interaction, and documentation of the behaviors and actions surrounding cowpea production, consumption, marketing and processing amongst others, this method assisted us in describing and interpreting the beliefs, values, roles, norms or behaviors as well as constraints and opportunities related to these issues on specific gendered groups within the cowpea value chain.

## 3 Results

**Figure 2:**
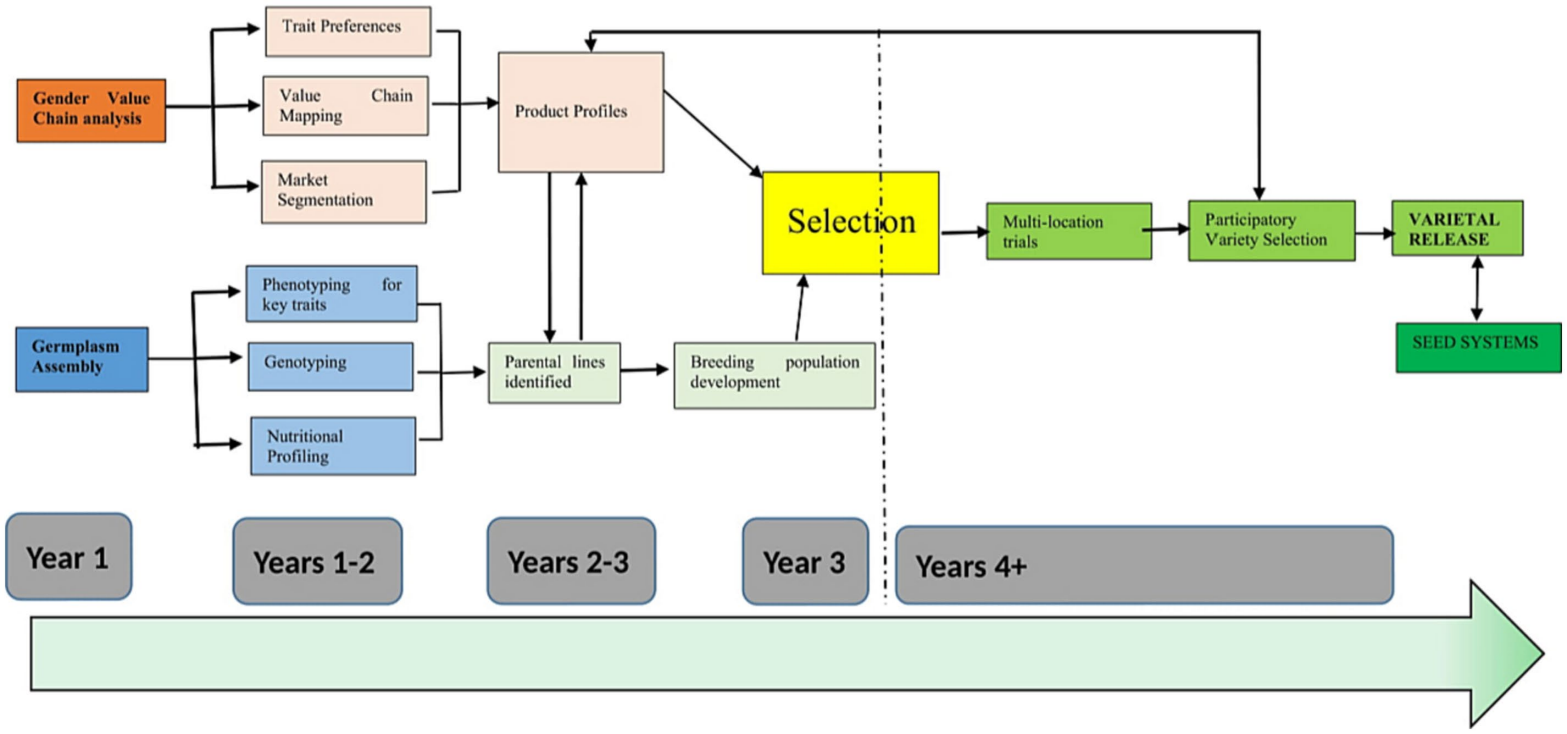
CICI-ESA gender sensitive cowpea breeding timeline.

Figure 2 depicts the gender-sensitive cowpea breeding timeline. The cowpea breeding programs under study in Malawi, Mozambique and Tanzania are relatively recent in terms of developing their own varieties. The current breeding initiative under the Center of Innovation for Crop Improvement for East and Southern Africa (CICI-ESA) was commissioned in 2021 with support from the Feed the Future Innovation Lab for Crop Improvement with a different approach of integrating gender. It had been noted that adoption levels of varieties that were introduced from outside the country and released in the respective countries were low. The gender research activities involved trait preference survey to understand farmers and consumers’ preferences for variety traits; cowpea value chain mapping to identify core processes of the chain (e.g., input supplies, production stages, postharvest and marketing stages), the enabling environment (infrastructure and policies, institutions and processes that shape the market environment and regulate the chain), value chain actors (identifying the chain actors, what they do, when and how, where and how the women and youth benefit, the flows of products in the chain, their volumes, values, and value addition at each step), service providers (i.e., input supplies, e.g., seeds, fertilizers), market information (e.g., prices, trends, buyers, suppliers, internal and cross border markets), financial services (e.g., credit, savings) and transport services (e.g., for grain purchasing); focus group discussions with men, women and youth farmers to have a deeper understating of the cowpea preferences; and key informant interviews with extension staff, local leaders, traders, agro dealers, processors to understand tradeoffs and price points.

The gender analysis study output at this early stage was meant to benefit breeders on how they can integrate gendered traits into mainstream breeding so that breeding programs are responsive to the needs of different gender groups so that varieties developed and released are taken up by different end users. Secondary beneficiaries of the gender study will be different end users, primarily farmers, consumers, traders, processors, researchers and policy makers. Lastly, the gender study will also aid in filling a knowledge gap in the literature that exists pertaining inclusive breeding process of improved cowpea varieties, in particular, as well as the broader scholarly debates in inclusive plant breeding more generally.

### 3.1 Cowpea value chain mapping in Malawi, Mozambique and Tanzania

**Figure 3:**
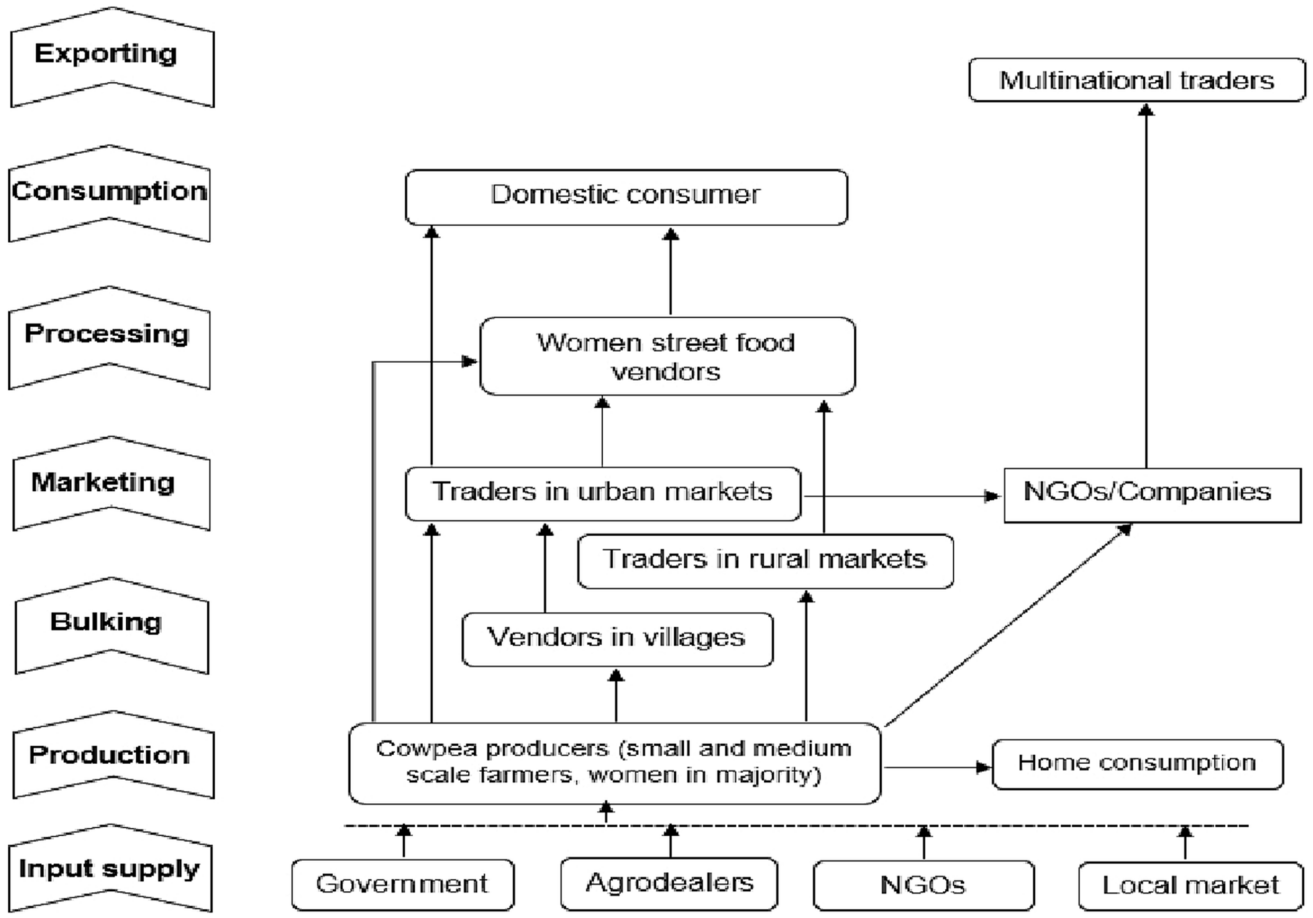
Schematic diagram of the cowpea value chain in Malawi from study.

**Figure 4:**
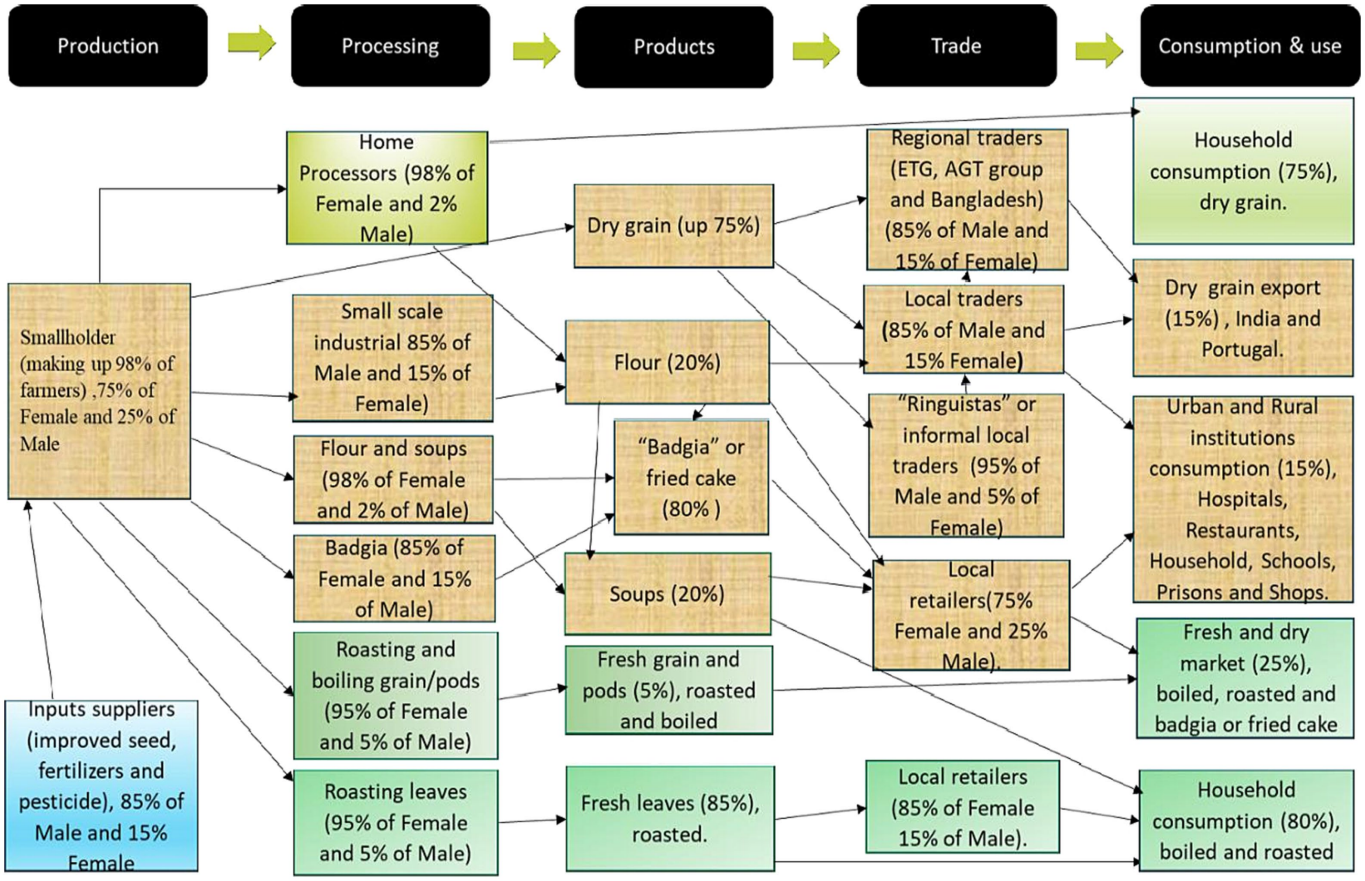
Example of a cowpea value chain map generated in Mozambique for the study.

From the value chain mapping, it was noted that major cowpea players included smallholder farmers (>98%), input suppliers (agro-dealers, government, non-governmental organisations), traders (aggregators), retailers, small scale home based processors and consumers. it was established that more than 75–80% of cowpea producers (the production node), are female farmers, while traders and agro-dealers constitute a range of 70 to 80% of male actors across the cowpea value chain within the sampled districts of the three countries (Figures 3, 4). Most of the cowpea is locally processed in these countries mainly at home into different products such as flour, fried cake (Bagia), soups, roasted, boiled, dried leaves, pulp locally known as *chipere* and 85% of players are females. Most of the cowpea and its products are consumed and utilised locally within these countries with few instances of exports to India, Portugal and other countries.

### 3.2 Cowpea traits preferences by gender

Tables 2–4 show trait rankings for Malawi, Mozambique and Tanzania, respectively. Overall, the top-ranking traits in order of importance across the countries and gender were; (1) high grain yield, (2) good grain taste, (3) early maturity time, (4) large grain size, (5) good leaf taste, and (6) short cooking time. It was further noted that different gender groups preferred almost similar traits though minor variations were noted in terms of prioritization of these traits. In Malawi, results showed that more adult males prioritised high grain yield, grain taste, field and storage pests’ resistance, disease resistance, drought tolerance and *Alectra vogelii* resistance than adult females. Similarly, more adult females prioritised traits such as short cooking time, early maturity, large grain size, leaf taste, leaf yield and grain color than adult males. In Mozambique, a similar trend was observed where a larger proportion of adult males than females preferred high grain yield, early maturity, grain size, drought tolerance, field and storage pests and disease resistance. More adult females than adult male preferred grain taste, short cooking time, leaf taste, leaf yield and grain color. For Tanzania, the proportion of adult females was larger than adult males for all traits.

**Table 2:** Ranking of some cowpea traits preferred by farmers and consumers across the six cowpea-producing districts in Malawi.

Trait	Gender	Ranking of cowpea traits in Malawi (%)				Overall rank
		Not important	Less important	Important	Very important	
High grain yield	AF	0.72	0.48	19.32	79.23	
	AM	0.55	1.38	12.4	85.12	1
	FY	2.59	2.59	18.1	76.72	
	MY	2.45	3.67	17.96	75.92	
Fast cooking time	AF	0.48	2.86	18.38	78.04	
	AM	1.15	7.2	29.68	61.67	2
	FY	3.86	4.29	22.32	69.53	
	MY	3.23	8.47	27.42	60.89	
Grain taste	AF	0.5	1.73	30.69	67.08	
	AM	0.55	2.47	25.82	71.15	3
	FY	2.15	6.87	26.61	64.38	
	MY	1.99	7.17	27.49	62.55	
Early maturity period	AF	0.73	7.06	28.47	63.5	
	AM	1.12	5.03	32.68	60.89	4
	FY	4.68	6.38	25.96	62.98	
	MY	3.24	8.5	25.91	62.35	
Large grain size	AF	0.74	6.14	26.29	66.83	
	AM	1.95	8.36	26.18	63.51	5
	FY	5.49	4.22	30.8	59.49	
	MY	2.82	8.47	29.84	58.47	
Leaf taste	AF	0.74	3.96	26.49	68.81	
	AM	1.65	11.54	29.95	56.87	6
	FY	3	8.15	25.32	63.52	
	MY	5.18	9.56	26.69	58.57	
Grain colour	AF	0.97	5.35	25.06	68.61	
	AM	1.7	11.36	27.84	59.09	7
	FY	4.72	5.15	27.9	62.23	
	MY	2.79	9.16	31.08	56.57	
Field pest resistance	AF	3.39	8.23	26.39	61.99	
	AM	2.81	7.02	28.93	61.24	8
	FY	5.15	12.02	22.32	60.09	
	MY	5.24	8.87	24.6	61.29	
Disease resistance	AF	3.4	8.98	25.97	60.92	
	AM	2.56	7.1	26.42	63.35	9
	FY	5.93	10.17	25.42	58.47	
	MY	3.21	10.44	26.1	60.24	
Storage pest resistance	AF	4.14	7.54	31.87	56.45	
	AM	3.91	8.38	28.49	58.94	10
	FY	5.15	9.87	24.03	60.94	
	MY	4.4	8.4	26.4	60.4	
Drought tolerance	AF	7.33	8.56	28.36	55.5	
	AM	6.63	6.91	25.97	59.67	11
	FY	8.58	10.73	18.88	61.37	
	MY	9.2	6	27.2	57.6	
Leaf yield	AF	0.98	8.78	29.27	60.98	
	AM	2.27	14.45	30.31	52.97	12
	FY	4.72	7.3	27.9	59.66	
	MY	5.6	10.8	28	55.2	
Growth habit	AF	1.95	9.73	30.66	57.42	
	AM	1.69	8.71	32.3	57.02	13
	FY	5.96	9.36	26.81	57.87	
	MY	4.45	12.15	28.34	54.66	
*Alectra vogelli* resistance	AF	15.35	21.1	10.55	53	
	AM	11.97	21.94	11.11	54.7	14
	FY	16.81	18.97	12.5	51.72	
	MY	13.94	21.12	12.75	52.19	

KEY: AF, adult female; AM, adult male; FY, female youth; MY, male youth.

**Table 3:** Ranking of some cowpea traits preferred by farmers and consumers across the six cowpea-producing districts in Mozambique.

Traits	Gender	Ranking of the cowpea traits (%)				Trait ranking
		Very important	Important	Less important	Not important	
Grain yield	AM	86.33	11.39	0.91	1.37	
	AF	81.74	15.07	1.37	1.83	
	MY	78.44	14.91	4.59	2.06	1
	FY	79.36	13.3	5.05	2.29	
Maturity period	AM	62.24	32.27	2.75	2.75	
	AF	61.24	31.65	2.52	4.59	2
	MY	61.66	26.79	5.31	6.24	
	FY	55.05	22.36	6.73	15.87	
Grain taste	AM	58.22	37.9	1.83	2.05	
	AF	62.93	32.95	1.6	2.52	3
	MY	57.47	34.48	5.06	2.99	
	FY	60.69	32.64	3.68	2.99	
Size of the grain	AM	58.9	29.91	3.88	7.31	
	AF	58.35	30.66	3.2	7.78	4
	MY	58.06	26.73	5.3	9.91	
	FY	57.14	23.49	8.47	10.9	
Leaf taste	AM	50.8	37.3	9.84	2.06	
	AF	55.73	35.09	7.11	2.06	5
	MY	49.66	32.87	10.8	6.67	
	FY	52.87	33.33	7.36	6.44	
Drought tolerance	AM	53.09	17.85	5.72	23.34	
	AF	52.29	17.66	6.19	23.85	5
	MY	52.87	17.01	6.21	23.91	
	FY	50.8	18.16	6.9	24.14	
Field pest resistance	AM	53.65	21.23	3.42	21.69	
	AF	52.86	21.28	3.66	22.2	6
	MY	51.61	20.87	4.59	22.94	
	FY	50.8	21.38	4.37	23.45	
Cooking time	AM	47.2	38.7	5.8	8.3	
	AF	51.96	39.26	1.62	7.16	
	MY	50.23	34.49	6.25	9.03	7
	FY	56.71	31.94	3.47	7.87	
Storage pest resistance	AM	51.26	22.2	5.03	21.51	
	AF	48.39	23.39	5.96	22.25	8
	MY	44.37	20.92	8.05	26.67	
	FY	42.53	21.15	9.2	27.13	
Tolerance to diseases	AM	45.89	23.52	6.62	23.97	
	AF	45.77	22.2	6.86	25.17	9
	MY	47.25	20.64	6.88	25.23	
	FY	46.56	20.87	7.11	25.46	
Grain colour	AM	40.96	36.61	8.47	13.96	
	AF	43.35	33.26	8.49	14.91	10
	MY	37.53	28.44	20.51	13.52	
	FY	41.94	32.53	9.68	15.86	
Leaf yield	AM	32.11	33.72	9.86	24.31	
	AF	41.51	26.61	9.63	22.25	11
	MY	33.33	28.97	10.34	27.36	
	FY	40.78	23.73	9.22	26.27	
Plant growth habit	AM	25.17	40.96	5.49	28.38	
	AF	23.39	41.51	6.19	28.9	12
	MY	25.06	37.7	7.82	29.43	
	FY	24.83	36.78	9.66	28.74	
Alectra vogelii resistance	AM	19.91	35.24	12.36	32.49	
	AF	18.35	33.26	14.22	34.17	13
	MY	19.35	31.34	14.52	34.79	
	FY	19.59	27.42	17.51	35.48	

KEY: AF, adult female; AM, adult male; FY, female youth; MY, male youth.

**Table 4:** Ranking of some cowpea traits preferred by farmers and consumers across the six cowpea-producing districts in Tanzania.

Trait	Gender	Ranking of cowpea traits in Tanzania (%)				Trait ranking
		Very important	Important	Less important	Not important	
Grain yield	AM	49.6	33.3	3.3	0.4	
	AF	78.3	19.2	0	0	1
	MY	20	27.9	4.2	1.3	
	FY	23.8	30	1.3	1.7	
Size of grain	AM	31.7	42.5	10	1.7	
	AF	56.3	34.6	5.4	0.8	2
	MY	11.7	27.5	9.2	2.5	
	FY	20.4	28.8	5.4	1.3	
Maturity period	AM	34.6	42.1	5.8	1.7	
	AF	50.8	36.7	7.1	2.1	3
	MY	15	24.2	7.5	2.5	
	FY	19.2	27.5	3.3	2.1	
Grain taste	AM	25.8	47.9	9.6	4.2	
	AF	65	30.8	1.3	0.4	4
	MY	4.2	34.6	13.8	2.9	
	FY	20.8	32.1	3.8	1.7	
Leaves taste	AM	20	35	22.5	7.9	
	AF	53.8	36.3	5.8	1.7	5
	MY	4.2	15.8	24.2	7.5	
	FY	15	35.8	5	2.5	
Cooking time	AM	16.3	37.9	14.6	9.2	
	AF	49.6	39.2	6.7	0.8	6
	MY	3.8	15.4	14.6	7.5	
	FY	20	25	6.3	2.9	
Drought tolerance	AM	19.2	46.7	13.8	2.5	
	AF	33.8	52.5	10.4	0.4	7
	MY	7.9	21.3	14.2	4.2	
	FY	6.7	29.2	10.8	4.2	
Leaf yield	AM	9.6	35	20.4	15	
	AF	37.1	40.4	15.4	2.1	8
	MY	2.9	12.5	17.1	11.3	
	FY	15.4	22.5	7.1	5	
Field pest resistance	AM	17.1	45.4	12.1	3.3	
	AF	30	46.3	17.1	0.8	9
	MY	5.4	22.9	13.8	3.3	
	FY	4.2	27.5	12.1	3.8	
Tolerance to diseases	AM	12.5	41.3	13.3	3.3	
	AF	29.6	37.1	16.7	5.4	10
	MY	4.6	18.3	7.1	9.2	
	FY	7.5	17.9	10	8.8	
Plant growing habit	AM	10.8	44.6	12.1	10.8	
	AF	25.4	53.3	13.3	2.5	11
	MY	5	19.2	12.1	4.6	
	FY	10.4	22.9	10.8	3.8	
Storage pest resistance	AM	14.2	43.8	15	7.5	
	AF	25.8	42.9	17.9	7.9	12
	MY	5.4	19.2	8.8	12.1	
	FY	5.4	21.7	10	10.4	
Grain color	AM	9.2	27.1	25	18.8	
	AF	20.8	48.3	19.2	7.5	13
	MY	4.2	15.8	18.8	9.2	
	FY	10	27.5	10.8	4.2	
Alectra vogelii resistance	AM	0	2.9	2.9	20.8	
	AF	0.4	5.4	4.2	29.2	14
	MY	0.4	3.3	2.9	7.1	
	FY	0	2.1	3.3	9.6	

KEY: AF, adult female; AM, adult male; FY, female youth; MY, male youth.

## 4 Discussion

The gender study results guided the breeding program in the identification of key priority traits. For example, the breeding program identified high grain yield and quality traits such as fast cooking time, taste and leaf taste as key priority traits for women and youth, while high grain yield, early maturity and pests and disease resistance as key priority traits for men in the three countries. Based on the priority traits, the objectives of the breeding program were realigned to incorporate key priority traits. Furthermore, the gender study helped in market/consumer segmentation which in turn helped in the creation of product profiles for each market segment.

### 4.1 Gender and breeding for target market(s) or end users

As a breeding program, the attention is on designing and developing specific new varieties to serve the needs and preferences of a specified group of customers with projected uptake of the varieties by different end-users. The ultimate end users of the cowpea varieties are farmers and consumers, both men and women. Farmers produce cowpea for both home consumption and sale to the market. As seen from our methodology, we made it a point that our breeding program should be gender inclusive from the onset, starting from the formulation of breeding objectives and trait prioritization, unlike past initiatives, which were not very explicit on the extent of stakeholders’ involvement in the breeding cycle. To put this in context, national cowpea breeding programs in Malawi, Mozambique and Tanzania in the past were not actively involved in actual variety development but rather they relied on advanced lines introduced from IITA for national release. Thus, most of these varieties developed were not based on local needs and preferences. Consequently, their uptake and adoption of these varieties was reportedly very low in Malawi (10%), Mozambique (10.9%) and Tanzania (31.3%) (IFPRI, 2020). This underscores for the need of national breeding programs to set their own national breeding objectives anchored on local needs and preferences. Therefore, guided by the need to enhance the uptake of nationally developed varieties, the comprehensive gender-sensitive cowpea value chain analysis resulted in three significant changes in the cowpea breeding pipeline in the three countries which were:

#### 4.1.1 Identification of priority traits

The study revealed that male and female cowpea farmers prioritized traits differently. For example, adult and young male farmers were mainly interested in market-oriented traits such as grain yield, early maturity, taste, pests and disease resistance. Their reasoning was that early maturing varieties would mean having two cycles of crop harvest within the same rainfall period, and they can quickly bring to the market. But this early maturing variety should have good taste since most buyers are local consumers who have such preferences. Obviously, the variety should be high yielding to make more money from small pieces of land. Similarly, women and female youth prioritized high grain yield but quality traits such as fast cooking time, taste and leaf taste were highly important to them. As a breeding program, we currently prioritize these traits with the anticipation of better adoption levels of to-be-developed varieties. The traits being prioritized in the three breeding programs based on the current study were also mentioned by other previous studies (Chiulele et al., 2011; Hella et al., 2013) as traits preferred by farmers in these countries. However, their studies did not consider gender disaggregated preferences.

#### 4.1.2 Designing breeding objectives

Decisions about the targeted breeding objectives take into account information about gender as it has been shown from the study that cowpea is an important crop for both men and women. Even though breeders are mainly responsible for coordinating, facilitating and linking actors and audiences with diverse interests, setting breeding objectives has become a multi-disciplinary exercise involving breeders, gender experts, nutritionists, and socio-economists to deliver varieties that have value on the market. The current breeding objectives are therefore driven by gender dynamics through the inclusion of traits that address the needs of both men and women at national level, such as grain taste, cooking time, maturity and grain size. Due to the overlapping nature of priority traits for male and female cowpea farmers, we aligned our breeding program objectives by incorporating key priority traits that address the needs of both men and women, including the youth within cowpea farming communities. According to Polar et al. (2022) gender-responsive breeding does not mean a program develops varieties specifically for women, but taking on board the needs of both men and women. Though not explicitly stated by farmers, our objectives tend to be more encompassing by including some essential resilience traits into breeding objectives.

#### 4.1.3 Creation of product targets/market segments and specific product profiles

Orr et al. (2018) argued that plant breeding for resource-poor farmers, sellers, and processors requires a marketing approach which among others requires market segmentation and development of breeding product profiles showing trait preferences for end users. From the current study, the majority of cowpea producers and end users are women who are mostly resource constrained. As such, there is always a need to capture information about gender when deciding which market segment or end users to target in terms of variety development. The gender study helped us in market/consumer segmentation which in turn helped to create product profiles for each product target. The current approach in our breeding program is consistent with modern breeding practices where market segmentation and product profile development are integral to an effective plant breeding program (Kimani, 2017; Thiele et al., 2021). Tables 2–4 illustrate how trait ranking by gender and age influenced the overall traits that have contributed to distinct product targets to cater to the differentiated needs of both adult/young females as well as adult/young males.

For instance, the Malawi cowpea breeding program now has designed two product targets, each with a specific product profile, i.e., (1) *Boiled grain* (mostly to cater to income generation needs, and large-scale cowpea farmers – addressing the preferences mostly raised by adult/young males as well as adult females interested to venture into large scale cowpea farming), (2) *Dual purpose* (leaf and grain) – (mostly targeting household and food security needs as well as small scale/local marketing – addressing most of the preferences raised by young/adult females). These differentiated product profiles stem from the extensive data (both qualitative and quantitative), where for instance, the first four preferred ranked traits for adult female farmers were cooking time, grain yield, grain color, and leaf taste, respectively. To also quote:

“…For women, they mostly grow cowpea for home consumption since they can use both its leaves and grain as relish throughout the year… As for men, they might assist their wives in some activities to do with cowpea production because it would help them have food stocks within their household. However, in cases where cowpea fetches higher prices, like the small, seeded ones in our area, you find men being involved more in cultivating such varieties than those that are preferred more for consumption in their homesteads…” (KII with Chiefs in Salima: 01/11/2021).

“…we usually prefer growing local varieties left by our great grandmothers/fathers due to easy access of seeds that are passed from one generation to another – but also because these varieties allow us to consume cowpea leaves and green pods throughout the year. So, we rely on such cowpea varieties in lean times, especially during periods when even finding relish, is not a simple task…” (FGD with Adult females-Karonga: 26/10/2021).

Similarly, Tanzania cowpea breeding program has also developed two product targets with specific product profiles based on their gender analysis study. These product profiles are meant to address cowpea gendered preferences for their farmers. Thus, based on the information on segmentation and gender disaggregated trait preferences in Table 4, Tanzania, like Malawi has developed two product targets to meet the needs of the farmers for boiled grain (targeting income generation and marketing) and dual-purpose cowpea (targeting home consumption and marketing).

For Mozambique, three product targets with specific product profiles have been developed to meet the needs of three market segments. Thus, the first one is for boiled grain with characteristics of short duration, brown colored seeds, large seed, nutritious, drought and disease tolerant for dry grain market targeting both male and female farmers (producers), processors, traders and consumers. The second one targeting both local markets (seed and grain) and international market (grain) is characterized by short duration, brown colored seeds, large seed, nutritious, drought and disease tolerant for dry grain market. The third one characterized by medium duration with brown and/or colored seeds, nutritious, drought and disease tolerant for dual purpose (leaves and dry grain) targets local markets (seed, leaves and grain) and international market (grain) for food and income generation.

Dual purpose product profiles thus target both home consumption needs and income generation by meeting market demands. A similar observation was noted in Senegal where they recommended that breeding programs need to prioritize breeding for dual-purpose cowpea varieties (Mukerjee et al., 2023). This stems from the fact that cowpea is a multipurpose crop and farmers can use it to address different needs ranging from human consumption, livestock feed to environment stewardship (Timko and Singh, 2008; Boukar et al., 2016; Abebe and Alemayehu, 2022). For those product profiles specifically being developed for international markets like the case of Mozambique, they specifically meet demands of male farmers, even thought female farmers also participate in marketing of such varieties at local level.

Across the three countries, we note substantial similarities in terms of trait preferences and prioritization among gender groups. These similarities might be due to similar characteristics among farmers and consumers in terms of farming systems, cowpea utilisation, marketing systems, types of cowpea varieties used (majority use local varieties with similar genetic characteristics). There is also a lot of cross border trades among these countries and perhaps farmers in respective countries produce cowpea targeting cross border markets and this in turn may also explain the apparent similarities in terms of trait preferences. This also implies that a desirable variety developed and released in any of these countries can easily be made available and adopted by farmers among these countries. This is in line with SADC Harmonized Seed Regulatory System (SADC Seed Centre, 2022) in ensuring that farmers have access to more high-quality improved varieties.

### 4.2 Lessons learned from the gender study

The gender-sensitive cowpea value chain study described here shows that gender analysis is very critical in designing a gender-responsive breeding program that is more likely to benefit both men and women. This study presented both opportunities and challenges to the breeding team. It was a remarkable opportunity to bring together researchers from very different fields and develop a common understanding of how breeding research could be designed and run.

The study brought to light that stakeholder engagement is key in developing product profiles as you reach a consensus on key traits for the breeding program. The trait preference survey revealed that trait ranking differs by gender, meaning that trait prioritization differs among these. This might present a challenge to breeders in terms of developing product profiles and varieties for different market segments with competing interests. Though trait ranking was somewhat different between men and women, it was also observed that the traits preferred were similar, and this kind of like provides a leeway for breeders to design varieties with product profiles that are more encompassing but realistic.

Working in a multi-disciplinary team needs patience and accommodating the procedures and methodological processes from the different fields involved. The Gender Value Chain Analysis being socially based, other uncontrolled factors meant that the breeding timelines had to be delayed due to such unforeseen circumstances. Since breeding protocols in sub-Saharan/resource-constrained countries are usually based on rainfed trials, the breeders had to adjust their timeline expectations based on the limitations faced by social scientists during data collection and analysis processes.

Training (of data collectors/enumerators) is key to successful and accurate data collection. It was noted that some enumerators had challenges in using some of the study tools for data collection. For example, some enumerators found it difficult to conduct choice experiments with farmers and consumers due to their technicalities and complexities. Also, some questions to respondents during the interviews were not properly phrased for the less educated respondents.

In terms of the technical team, it may help to accept from the onset that gendered breeding is a continual process. It is, therefore useful to set timed milestones and aim for the best synthesis of data and information within the available time. We found that data analysis and insight generation kept getting better with time. However, once we hit an acceptable threshold of insight to improve on past breeding initiatives substantially, the team decided to move on. Thus, we find that gendered breeding presents an ongoing need and challenge for continual improvements in methods and protocols such that more time, attention and detail are provided for. This also echoes Chambers’ (2008) in speaking to the need to start early and proceed methodically with a solid team.

In the same vein, the bringing together of team members from varied technical fields means that a critical success factor is to facilitate the overlap of their technical siloes for meaningful conversations. Brainstorming sessions, idea-sharing sessions, learning workshops, frequent-enough meetings, team building efforts, and other activities are helpful for the team members to begin to understand each other’s perspectives, to speak to each other empathetically, get acquainted with resources available to the team as well as, very importantly, to understand their respective and joint roles in the program clearly. The social economist necessarily must understand clearly enough how their input feeds into the ultimate—breeding process. The plant breeder must similarly understand the methods available to the social scientist, including their limitations and correct interpretation of processes and results. This helps the breeder in guiding and inputting into the synthesis of data for market segmentation and product profiling, for example.

### 4.3 Challenges and study limitations

Considering the breadth and depth of the data collected in the gender-sensitive value chain analysis study, several challenges and limitations must be considered in similar studies conducted towards inclusive breeding ends.

One challenge that confronted our breeding program was the high attrition of social economists. We changed at least two economists before finding a stable substitute. This meant that the substitute had missed an important team-building and project familiarization period at the start of the project. He thus came into a steep learning curve whilst we were heading out for the value chain analysis survey, impeding how much input he could make at the time. For example, he later observed that a cowpea demand study would have been useful; but it was too late by then to incorporate this component in the program’s current phase. We plan to incorporate this in successor phases or projects.

Research assistants had difficulty clarifying the names of local cowpea varieties since the names were either given based on use, growth system or how they were introduced in specific areas. As such, most of the local varieties present were named differently within different settings, even if they had similar characteristics.

Most of the research assistants who collected data were either ongoing undergraduates and a few graduate students, a deliberate choice that was made by the research team to build capacities as part of the CICI-ESA project goals. However, this contributed to some study limitations since most of them had difficulties initially employing choice experiments and qualitative data transcription and reporting, despite the team being rigorously trained. Nonetheless, this challenge was rectified through a rigorous data-cleaning process as well as further transcription of audio recordings during the qualitative analysis process.

## 5 Conclusions and recommendations

Our study was able to identify key cowpea traits that are gender, youth, and resilient inclusive and these have been critical to informing the development of specific cowpea product profiles and market segments. Much as it was a rigorous process, the team recommends that it is a worthwhile and critical process to undertake for any inclusive plant breeding initiative. Plant breeders might not necessarily have to produce specific breeding lines for each gender group, but they can come up with product profiles that can cater to several needs and differences that are unveiled through a gender-sensitive breeding process.

Being designed as comparative research across three countries, i.e., Malawi, Tanzania and Mozambique, collaborative breeding efforts that are based on a comprehensive value chain analysis allow researchers to map out actors and connections beyond the borders, hence creating an opportunity to map out market demands that can be beneficial for farmers beyond their farming locations.

Having a multidisciplinary team to achieve inclusive breeding agenda pays off in the end. However, there is a need for the team members to be able to embrace the following principles: Shared/Common Goals, Flexibility; Co-Learning; Co-Creation; Mutual respect and accountability. This is because there should be a realization that all stakeholders participating at each level of the process, are critical to achieving your set goals.

## Data availability statement

The original contributions presented in the study are included in the article/supplementary material, further inquiries can be directed to the corresponding author.

## Author contributions

MC: Conceptualization, Funding acquisition, Investigation, Supervision, Writing – original draft. JK-P: Investigation, Methodology, Writing – review & editing, Supervision. DM: Formal analysis, Methodology, Writing – review & editing, Data curation, Investigation. HC: Investigation, Supervision, Writing – review & editing. MT: Investigation, Project administration, Supervision, Writing – review & editing. DB: Investigation, Supervision, Writing – review & editing. JH: Investigation, Supervision, Writing – review & editing. EY: Investigation, Writing – review & editing. NM: Investigation, Writing – review & editing. JK: Project administration, Data curation, Formal analysis, Writing – review & editing.
